# *Journal of Foot and Ankle Research* reviewer acknowledgement 2015

**DOI:** 10.1186/s13047-016-0136-7

**Published:** 2016-02-01

**Authors:** Alan Borthwick, Hylton Menz

**Affiliations:** University of Southampton, Southampton, UK; La Trobe University, Bundoora, Australia

## Abstract

The editors of *Journal of Foot and Ankle Research* would like to thank all our reviewers who have contributed to the journal in Volume 8 (2015).

**Muaaze Ahmad**

United Kingdom

**Paul Armanasco**

Australia

**Toni Arndt**

Sweden

**John Arnold**

Australia

**Thais Aurichio**

Brazil

**Stuart Baird**

United Kingdom

**Shan Bergin**

Australia

**Chris Bishop**

Australia

**Alison Blake**

United Kingdom

**Daniel Bonanno**

Australia

**Virginia Bower**

Australia

**Elizabeth Brainerd**

United States of America

**Ivan Bristow**

United Kingdom

**Andrew Buldt**

Australia

**Felix Burden**

United Kingdom

**Joshua Burns**

Australia

**Paolo Caravaggi**

Italy

**Matt Carroll**

New Zealand

**Paul Chadwick**

United Kingdom

**Lindsey Cherry**

United Kingdom

**Nachiappan Chockalingam**

United Kingdom

**Richard Collier**

United Kingdom

**Mark Cornwall**

United States of America

**Matthew Cotchett**

Australia

**Stephen Cousins**

Australia

**Emma Cowley**

United Kingdom

**Mary Cramp**

United Kingdom

**Gillian Crofts**

United Kingdom

**Michael Curran**

United Kingdom

**Brian Ellis**

United Kingdom

**Angela Evans**

Australia

**Lisa Farndon**

United Kingdom

**Gwen Fernandes**

United Kingdom

**Malindu Eranga Fernando**

Australia

**Jill Ferrari**

United Kingdom

**Robert Field**

United Kingdom

**Martin Fox**

United Kingdom

**Lucy Gates**

United Kingdom

**Alfred Gatt**

Malta

**Dominic Gehring**

Germany

**Moshi Geso**

Australia

**Kellie Gibson**

United Kingdom

**Nikolaos Gougoulias**

United Kingdom

**Andrea Graham**

United Kingdom

**Kelly Gray**

Australia

**Rodney Green**

Australia

**Martin Gronbech Jorgensen**

Denmark

**Jill Halstead**

United Kingdom

**Joseph Hamill**

United States of America

**Farina Hashmi**

United Kingdom

**Gordon Hendry**

United Kingdom

**C. Collin Herb**

United States of America

**jj Hermans**

Netherlands

**Claire Hiller**

Australia

**Brian Horsak**

Austria

**Sheree Hurn**

Australia

**Kasper Janssen**

Netherlands

**Sara Jones**

Australia

**David Kachlik**

Czech Republic

**Anne-Maree Keenan**

United Kingdom

**Robert Kidd**

Australia

**Tim Kilmartin**

Ireland

**Dorit Kunkel**

United Kingdom

**Toshiyuki Kurihara**

Japan

**Peter Lazzarini**

Australia

**Alberto Leardini**

Italy

**Pazit Levinger**

Australia

**Kirsten Leyland**

United Kingdom

**Anmin Liu**

United Kingdom

**Vincent Luboz**

France

**John Ludlow**

United States of America

**Ryan Mahaffey**

United Kingdom

**Matthew Malone**

Australia

**Michelle Marshall**

United Kingdom

**Joan Marti**

Spain

**Ian Mathieson**

United Kingdom

**Marlene Mauch**

Switzerland

**joanne McCardle**

United Kingdom

**Keith McCormick**

United Kingdom

**Julie McDonald**

Australia

**Karen Mickle**

Australia

**Anna Moran**

Australia

**Stewart Morrison**

United Kingdom

**George S Murley**

Australia

**Vanessa Nube**

Australia

**Simon Otter**

United Kingdom

**Kade Paterson**

Australia

**Smadar Peleg**

Israel

**Byron Perrin**

Australia

**Jill Phethean**

United Kingdom

**Stephen Preece**

United Kingdom

**Carina Price**

United Kingdom

**Trevor D Prior**

United Kingdom

**Anita Raspovic**

Australia

**Michael Skovdal Rathleff**

Denmark

**Anthony Redmond**

United Kingdom

**William Ribbans**

United Kingdom

**Jody Riskowski**

United Kingdom

**Edward Roddy**

United Kingdom

**Keith Rome**

New Zealand

**Dominique Rouleau**

Canada

**Dale Schuit**

United States of America

**Debbie Sharman**

United Kingdom

**Heidi Siddle**

United Kingdom

**Martin Spink**

Australia

**Kate Springett**

United Kingdom

**Michelle Spruce**

United Kingdom

**David Stephensen**

United Kingdom

**Martijn Steultjens**

United Kingdom

**Simon Taylor**

Australia

**Scott Telfer**

United Kingdom

**Masafumi Terada**

United States of America

**Jo Tweed**

United Kingdom

**Stephen Urry**

Australia

**Jaap van Netten**

Netherlands

**Edgar Vieira**

United States of America

**Scott Wearing**

Australia

**Caleb Wegener**

Australia

**Michael Wilding**

Singapore

**Richard Wilkins**

United Kingdom

**Cylie Williams**

Australia

**Marinus Winters**

Netherlands

**James Woodburn**

United Kingdom

**Gavin Wylie**

United Kingdom

**Michael Yelland**

Australia

**Maria Young**

United Kingdom

**Bernhard Zipfel**

South Africa

